# Social Presence, Negative Emotions, and Self-Protective Behavioral Intentions of Nonsmokers in Response to Secondhand Smoking in Virtual Reality: Quasi-Experimental Design

**DOI:** 10.2196/46243

**Published:** 2023-10-25

**Authors:** Miao Liu, Yicheng Zhu, Zihan Xu, Sitong Meng

**Affiliations:** 1 School of Journalism and Communication Beijing Normal University Beijing China

**Keywords:** virtual reality, VR, social presence, emotions, secondhand smoking

## Abstract

**Background:**

The application of virtual reality (VR) in health care has grown rapidly in China, where approximately half of the population is directly exposed to secondhand smoke (SHS). As VR headsets have become increasingly popular and short video platforms have incorporated 360° videos in China, new formats and opportunities for health campaigns about SHS have emerged.

**Objective:**

In a simulated environment of exposure to SHS, this study aims to explore the emotional and behavioral responses to enhanced social presence brought about by VR in contrast to flat-screen videos. It also aims to examine whether and to what extent video modality (360° video vs flat-screen video) and contextual cues (high threat vs low threat) influence psychometric and intentional variables among viewers.

**Methods:**

A total of 245 undergraduate and graduate students who were nonsmokers and from a large university in China participated in this study between October 2020 and January 2021. This study created 4 different versions of a SHS experience in a café with a 2 (360° video on a head-mounted display vs flat-screen display) × 2 (high threat vs low threat) experimental design. It developed and tested a path model examining the effects of experience modality and threat levels on social presence, emotions (anger and disgust), and eventually behavioral intentions (staying away and asking for help).

**Results:**

We found that both video modality (*P*<.001) and threat level (*P*=.005) significantly influenced social presence, whereas the interaction of video modality and threat level did not have a statistically significant effect on social presence (*P*=.55). Negative emotions mediated the relationships between social presence and SHS-related self-protective behaviors. Specifically, anger positively predicted the intention to ask smokers to stop smoking through the waitress (*P*<.001). Disgust and fear both positively predicted the intention to stay away from the SHS environment (*P*<.001 for disgust; *P*=.002 for fear).

**Conclusions:**

This study explored the potential mediating mechanisms that influence individuals’ responses to the risks of SHS in public areas. The results demonstrated that social presence and negative emotions are 2 important mediators that underlie the relationship between video modality and behavioral intention regarding SHS in a VR setting. These findings suggest that an immersive environment could be a better stimulator of anti-SHS emotions and behaviors than flat-screen videos.

## Introduction

### Background

Secondhand smoke (SHS) is a severe yet understudied health issue in China. SHS could cause an increased risk of respiratory illness, heart disease, lung cancer, and asthma and worsen chronic obstructive pulmonary disease [1]. According to the Global Adult Tobacco Survey China, more than half of the Chinese people reported that they were exposed to SHS in the workplace and 44.9% of the respondents were exposed to SHS at home [2]. Data from the World Health Organization revealed that >700 million nonsmokers are exposed to SHS at least once a day in a typical week, and exposure to SHS causes 100,000 deaths annually in China [3]. Even though smoke-free policies prohibiting smoking in indoor public places came into force in certain metropolitan areas such as Beijing and Shanghai, SHS is still not rare in many restaurants, bars, and workplaces. The lack of implementation resources, together with a collectivist culture, makes it difficult to implement the smoking-free policies. The rate of exposure to SHS in public places is higher in China than in many other countries [4].

Antismoking mass media campaigns can effectively change smoking attitudes and beliefs, promote smoking cessation, and reduce adult smoking prevalence [5]. The 2006 Surgeon General’s report indicated that the only way to protect nonsmokers from SHS was to eliminate exposure from smoking [6]. However, there are insufficient health campaigns targeting the SHS problem in China, and the message effect of these health campaigns has not been extensively examined and evaluated. Moreover, current SHS control programs have been mainly targeted at smokers such as male smokers [7] and smoking parents [8,9], whereas less effort has been made to encourage SHS self-protective behaviors of nonsmokers. Considering the high prevalence of SHS in indoor public places in China, interventions that target motivating SHS self-protective behaviors in nonsmokers are worth investigating. The purpose of this study was to explore the delivery channel and the content of health messages that could influence individuals’ responses to SHS in public places.

### Health Campaigns in Virtual Reality: The Role of Presence and Context

Virtual reality (VR) is broadly defined as a computer-generated environment that allows users to sense and interact with other objects and people as if they are in the real world [10]. Among the various types of VR technology applications, immersive virtual environments (IVEs) can be experienced by users through head-mounted displays (HMDs) such as Oculus Rift, HTC Vive, or Pico Pro. As one of the modalities of IVEs, 360° videos have become a new health communication tool and have been increasingly incorporated into health education and health promotion campaigns [11-13]. In the context of health education, a meta-analysis revealed that an IVE is more effective than other types of digital education in increasing health professionals’ knowledge and cognitive skills [14]. Media messages that incorporate VR technologies can have a great impact on audiences’ personal risk perceptions and thus motivate healthy behaviors. The study by Ahn et al [11] found that 1 week after exposure, a pamphlet coupled with an IVE led to less soft drink consumption than the noncoupled message (just a pamphlet). An IVE has also been used as a mode of cue presentation in smoking cessation programs [15]. However, the underlying mechanism by which VR technologies promote specific health behaviors has received scant attention.

The application of IVEs in health-related campaigns was found to be effective when the viewer is situated as a first-person *participant*. For users of an IVE, different person perspectives (ie, observer, partaker, and victim) provide distinct levels of social interactability for the intradiegetic actors. As a third person, the avatar in the virtual environment usually lacks the ability to socially interact with characters and objects. However, a first-person “victim” perspective could provide the user with an experience similar to an extrafictional victim’s lived experience [16]. This study focuses on this first-person perspective of “victim” as it could most effectively position the user into the fictional experience of being harassed by SHS, where objects such as exhaled smoke or tossed cigarette butts could be oriented toward the user directly. As the study will review later, these fictional social interactions, along with the users’ feeling of being forcefully engaged with socially undesirable interactions, are critical in arousing negative emotions.

Social presence, simply defined as the “sense of being with another” [17], is essential in creating effective first-person IVEs. Social presence as an inherent consequence of the modalities with varying levels of immersion has been well accepted in the VR literature [18]. Conceptually, immersion can be a technological affordance of virtual environments that works as a catalyst for the enhancement of psychological presence [19]. Although others have conceptualized immersion as a subcomponent of a larger presence construct [20], the effect brought by modality change on presence is well established. In the current case, 360° videos experienced in HMDs can arguably create a higher level of isolation for the user from the external world. More importantly, health behavior experiments using 360° videos are theoretically more interested in how the intentions of social interactive behaviors are influenced. Hence, this study focuses on social presence as a key theoretical nexus, instead of adopting a multidimensional focus on the larger concept of presence [21].

Previous studies have shown that individuals in 3D model–based and 360° video–based immersive environments experience a higher level of social presence than individuals who use flat-screen displays [22,23] because the VR setup could provide a wider range of sensory channels [24]. Apart from technological modality, contextual properties such as social cues and agency also influence social presence [18]. Social presence could be influenced by the psychological processes through which individuals interpret available social cues in a positive or negative way [18]. Studies have suggested that cues that indicate the social context (eg, the number of people) could lead to a higher level of social presence [25,26]. Similarly, the study by Lombard and Ditton [27] suggested that content variables such as human and nonhuman characters influence presence. Attentional bias toward threat suggests that people are more prone to direct their attention to threatening stimuli than to neutral stimuli [28]. Empirical research has shown that a higher level of threat could induce a prevention focus [29]. According to the explication of social presence by Cummings and Wertz [30], involvement, which manifests as focus or attention, is an important dimension of social presence. In this study, we propose that threat level is a contextual cue that influences the level of social presence. A high level of threat is expected to elicit a higher level of social presence. Thus, we propose the following hypotheses:

- Hypothesis 1: participants viewing the content in 360° video format with HMD (vs flat-screen display) feel more social presence.
- Hypothesis 2: participants viewing the content with high threat feel more social presence than those viewing the content with low threat.

However, the interaction between video modality and threat level is less certain. Studies have shown that immersive media forms could enhance the effectiveness of risk communication [31,32]. In addition, health messages with a higher level of threat are generally more persuasive than those with a lower level of threat [33]. One study found that modality (immersive VR vs flat-screen display) modulated different types of threats [34]. The study by Jin and Brown-Devlin [35] found that perceived threat is a moderator that can maximize the effects of social presence. In summary, threat emerges as an important contextual factor that influences the effect of modality on social presence. To our knowledge, there is no extant research that directly examines the interaction effect between modality and threat level on social presence. Therefore, we propose the following research question: Is there an interaction effect between video modality and threat level on social presence?

### Social Presence and Emotion in the VR Setting

Generally, it has been found that IVEs have a stronger effect on eliciting emotions than the traditional flat 2D screen. The study by Jimenez et al [36] compared the player’s responses to emotional content displayed on VR and flat screens. It has been found that after experiencing both conditions, VR provides a more intense and frightening experience than playing on a traditional monitor. The study by Susindar et al [37] found that emotionally charged stimuli in IVEs can effectively elicit anger and fear. Social presence, as a crucial component in creating realistic IVE experiences, is a key mechanism that produces the effect of modalities on individuals’ emotional responses. Social presence is intrinsically linked to emotion. According to the study by Garrison et al [38], emotion is an indicator of social presence. The association between social presence and emotion has been demonstrated abundantly in VR research. A systematic review conducted by Diemer et al [39] showed that the association between presence and emotion has mainly been examined by means of correlations between these 2 measures. For example, 1 study found that socially anxious participants reported higher social presence than their peers in an interactive virtual environment [40]. Another study found a correlation between presence and fear in the text-anxious group in IVE [41]. Recent research has begun to explore the causal effect of social presence on emotional responses [42,43]. For example, it is found that social presence positively influenced empathy by making the user feel more comfortable in a virtual environment [44]. In health settings, the portrayal of the same antismoking message using spatial augmented reality statistically significantly influenced negative emotions through its effect on spatial presence compared with a 2D screen display [45]. On the basis of the previous literature, the following hypothesis is proposed:

- Hypothesis 3: social presence positively predicts negative emotions including anger (hypothesis 3a), fear (hypothesis 3b), and disgust (hypothesis 3c).

### Effects of Emotions on Behavior in SHS Scenarios

Emotion could trigger a set of responses (physiological, behavioral, experiential, and communication) that prompts individuals to address encountered problems quickly [46]. Negative emotions play a powerful role in influencing individuals’ self-protective behaviors [47-50]. Eliciting negative emotional responses has been increasingly used as an effective strategy in persuasive health messages. An ample number of prior studies have demonstrated that emotionally charged information about the adverse effects of smoking could effectively promote positive behavioral change. Research has shown that emotionally arousing antismoking advertising is more likely to be recalled and promote high-order cognitive processing [51,52]. The antismoking information that aroused the strong negative emotions of fear and sadness was rated as more effective by youth participants [53].

Emotion has its action tendency. Generally, emotions can be categorized based on approach and avoidance tendencies. The approach or avoidance motivation model of emotions provides a theoretical foundation for the causal effects of emotions on behavior [54]. In this study, we focused on 3 distinct negative emotions, including anger, fear, and disgust, which are often associated with exposure to SHS or viewing antismoking messages [55-58]. Anger, characterized by approach or agonistic motivation, is often elicited by negative or offensive stimuli [59,60]. Research has suggested that anger has a motivational tendency to subdue offending stimulus [61]. Fear is associated with avoidance tendencies and inhibition [60]. In media effect research, fear is often induced by threat appeal [62]. Fear has the function of motivating efforts for self-protection [61]. Similar to fear, disgust is an avoidance or withdrawal emotion. Disgust is often associated with the appraisal theme of being too close to a potentially contaminating object [63]. Disgust is characterized by motivating physical avoidance of a disgusted object or behavior [64].

**Figure 1:**
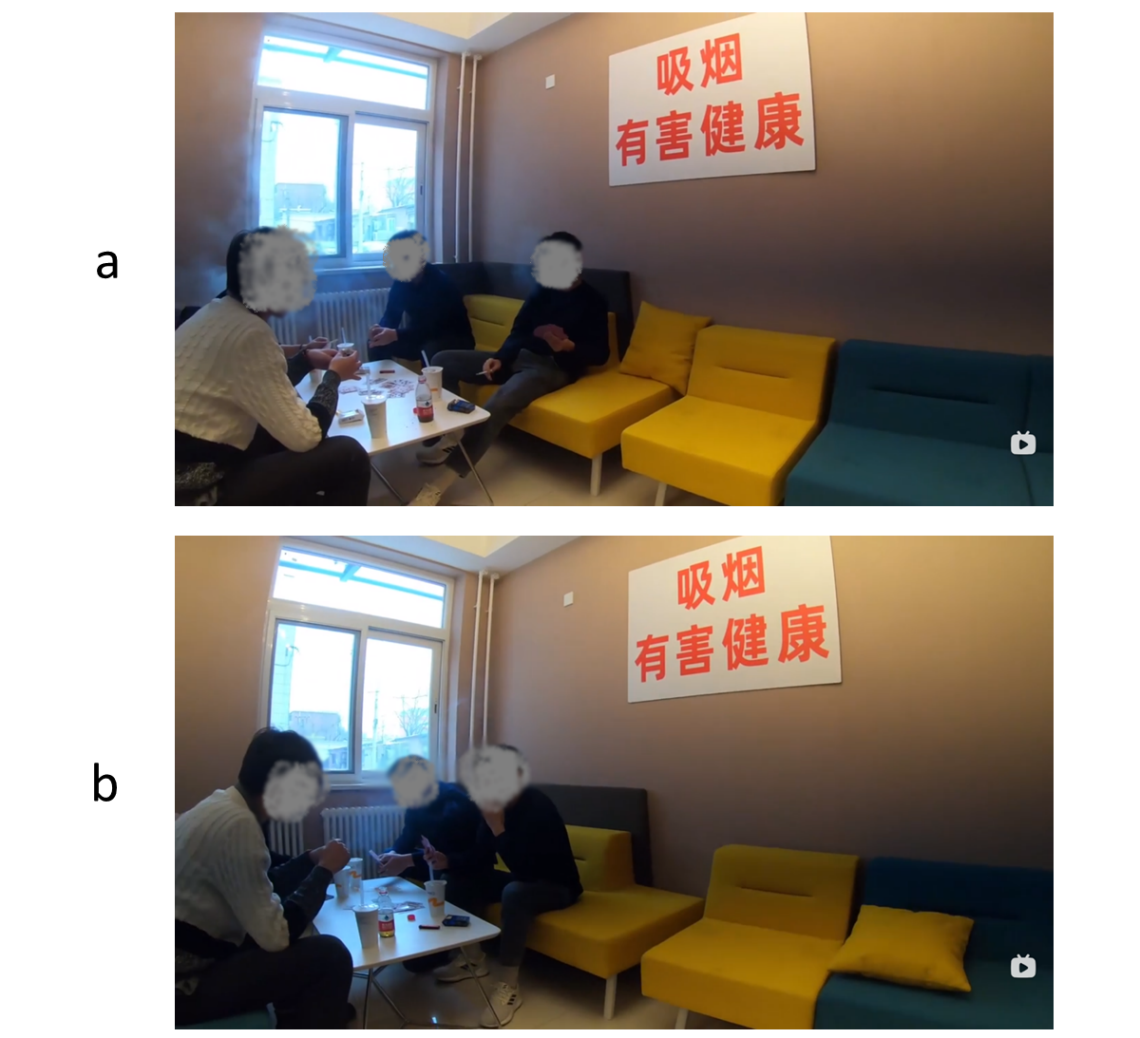
Screenshot of stimulus videos for flat-screen conditions: (A) high threat × flat-screen condition and (B) low threat × flat-screen condition.

Previous research has examined the effects of personal traits and behavioral factors on SHS avoidance behavior [65]. In this study, we focused on the emotional antecedents of SHS avoidance behaviors. On the basis of a previous theoretical framework, anger, as a negative approach emotion, is hypothesized to predict the intention to ask smokers not to smoke through the waitress. Fear and disgust, both avoidance emotions, were hypothesized to predict the intention to stay away from SHS. The conceptual models are depicted in Figure 1A (model 1) and Figure 1B (model 2).

- Hypothesis 4: anger positively predicts the behavioral intention of asking smokers to stop smoking through the waitress.
- Hypothesis 5: fear and disgust positively predict behavioral intention to stay away from the SHS environment.

## Methods

### Participants and Design

A total of 245 undergraduate and graduate students who are nonsmokers and from a large university in China participated in this study between October 2020 and January 2021 (mean age 22.16, SD 13.08 y). Individuals with a history of smoking, even if they were currently nonsmokers, were excluded from this study. The design and conduct of the experiment were preapproved and foreseen by the research ethics review board at the institution. The research recruitment flyer was distributed through the most popular Chinese social app, WeChat. Female students accounted for 166 (67.8%) out of 245 participants, whereas male students accounted for 79 (32.3%) of the participants.

A 2 (360° video in HMD vs flat-screen display) × 2 (high threat vs low threat) factorial experiment was conducted on the web and in the laboratory setting. All the participants were randomly assigned to 1 of the 4 experimental conditions. The HMD conditions included 122 participants, with 58 and 64 participants in the high-threat and low-threat condition, respectively. The flat-screen conditions included 123 participants, with 60 and 63 participants in the high-threat and low-threat condition, respectively.

Participants assigned to the flat-screen condition were asked to complete a questionnaire on the Qualtrics (Qualtrics International Inc) website using a computer, and those assigned to the VR condition (360° video in HMD) were asked to come to the laboratory. To ensure that all the participants in the flat-screen condition watched the video using a computer, we disabled the mobile function on Qualtrics and added highlighted instructions that the videos should be viewed in full-screen mode. Before the experiment, the participants electronically signed a consent form. The Pico Goblin 2 headset was used to present stimuli in the VR condition. The participants could look around in the virtual environment for the VR conditions of the study. In the VR condition, the participants were debriefed upon completing the survey. All participants who completed the study were rewarded with approximately US $5.

Power analysis assuming an α level of .05 and a power of 95% suggested that a sample size of 212 was sufficient to detect a medium effect size of *f*=0.25.

### Message Manipulation

The experimental stimuli consisted of four 2-minute short videos created by the authors. The flat-screen stimulus was shot using a GoPro-4 camera, whereas the VR stimuli were shot using Insta 360 One X. All the videos were shot from the first-person perspective. Figure 1A depicts the mise-en-scène of the high-threat, flat-screen condition, and Figure 1B depicts the low-threat, flat-screen condition. The 360° videos were created using the same settings but with a 360° video camera. The HMD allowed viewers to view 360° videos under VR HMD conditions. At the beginning of the videos, the main character (the participant) was sitting in a café, where 4 men playing poker cards can be seen across the participant. In the high-threat condition, 4 smokers were smoking at the same time while playing poker cards. In the low-threat condition, there were 4 people playing poker cards but only 1 person was smoking in the video. Both the high-threat and low-threat stimuli were approximately equal in length. To ensure that the message content is the same across the 2 delivery channels, the experiment stimuli that had the same threat level were shot simultaneously.

### Measures

#### Behavioral Intentions Toward SHS

A total of 2 SHS-related behavioral intentions were measured in this study: asking smokers to stop smoking through the waitress (mean 3.73, SD 1.03) and leaving the SHS environment (mean 2.89, SD 0.69).

#### Negative Emotions

Negative emotions, including anger, fear, and disgust, were measured using a self-report measure of discrete emotions [66]. Disgust was measured by 3 items: disgusted, sickened, and grossed out (mean 4.12, SD 0.68; α=.69). Fear was measured using 3 items: fearful, afraid, and scared (mean 2.89, SD 0.86; α=.73). Anger was measured using 4 items: irritated, angry, annoyed, and aggravated (mean 3.87, SD 0.87; α=.85). Participants were asked, “While you were in the virtual reality environment/watching the video, to what extent did you experience each of the following emotions?” (0=“none of this feeling”; 4=“a great deal of this feeling”).

#### Social Presence

Social presence was measured using the social presence–actor scale, originally developed by Lombard et al [21]. A total of 4 items from the scale were adapted in a study examining first-person social presence, of which 1 example item asked, “How often did you have the sensation that people you saw/heard and also see/hear you?” (1=“never”; 7=“always”) [12]. We used the same items whose scores were then averaged to form an overall measure (mean 3.15, SD 1.31; α=.87).

#### Control Variables

To determine whether any control variable should be included in the path model, we performed a randomization check on the distribution of age (continuous), gender (dichotomous), family income (categorical), and whether the participant was living with a smoker (dichotomous) across experimental conditions. Results of chi-square tests (on gender, family income, and living with a smoker) and an ANOVA test (on age) showed that there were no distributional differences in these demographic variables for age, family income, and living with a smoker. Thus, no control variables were included in the path model.

### Manipulation Check

Perceived threat level was assessed by 1 question, “How much risk do you think is associated with SHS in the video you just watched?” (mean 3.95, SD 0.95).

### Analysis Strategy

Path analysis based on the maximum likelihood estimation was conducted using Stata (version 15; StataCorp). Model fit was assessed based on conventional criteria, including chi-square test, root mean square error of approximation (RMSEA), standardized root mean square residual (SRMR), and comparative fit index (CFI). A value of ≤0.06 for RMSEA, <0.09 for SRMR, and ≥0.95 for CFI can be considered as a good fit [67]; we performed path analysis based on 5000 bootstrap samples.

### Ethics Approval

The study was approved by the institutional review board of the School of Journalism and Communication at Beijing Normal University (protocol code BNUJ&C20200706001; July 6, 2020).

## Results

### Effect of Modality on Social Presence

**Figure 2:**
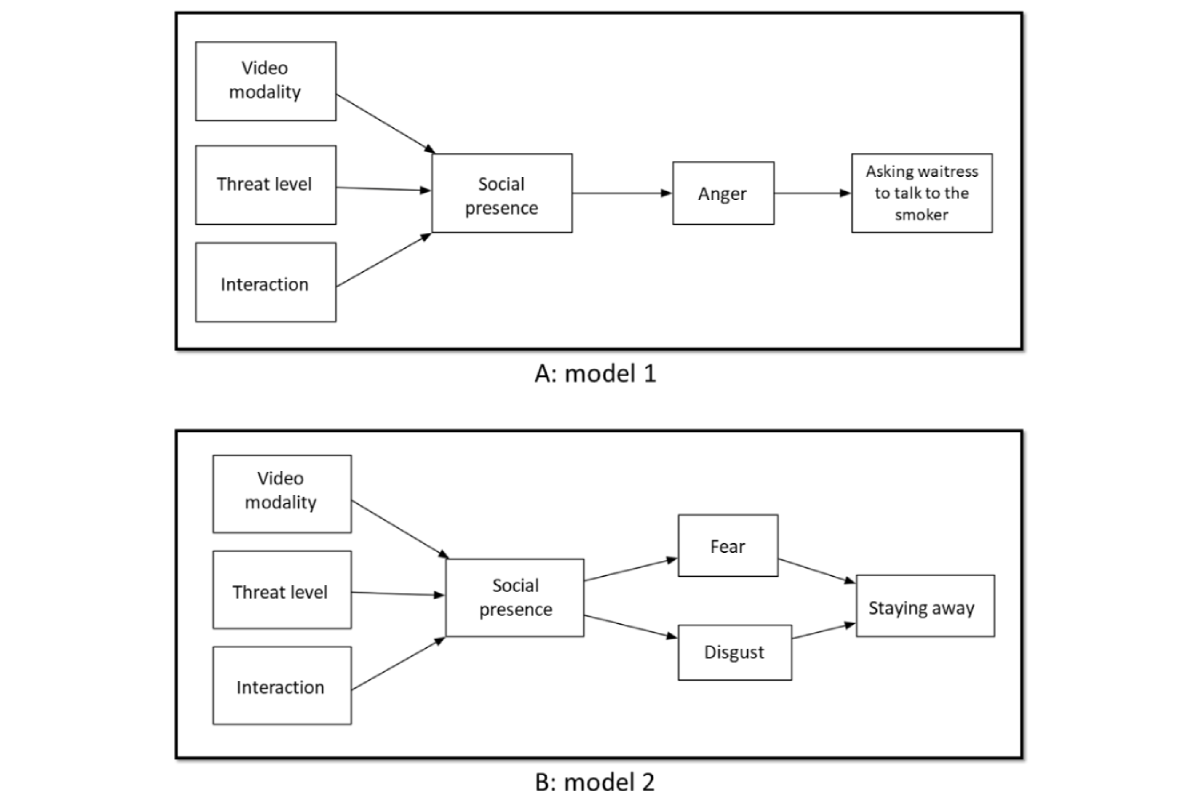
Proposed path models: (A) model 1 (with asking waitress as the outcome variable) and (B) model 2 (with staying away as the outcome variable).

The manipulation check was successful. A 2-way ANOVA was used to assess the effect of threat level on perceived risk. The threat levels induced statistically significantly different levels of perceived risk (*F*_1,244_=4.87; *P*=.02). Participants in the high-threat condition were more likely to perceive the environment to be riskier (mean=4.08, SE=0.08) than those who were in the low-threat condition (mean=3.82, SE=0.09). Table 1 shows the bivariate correlations between all the variables included in the path model. The proposed models depicted in Figure 2A and Figure 2B were tested using path analysis.

**Figure 3:**
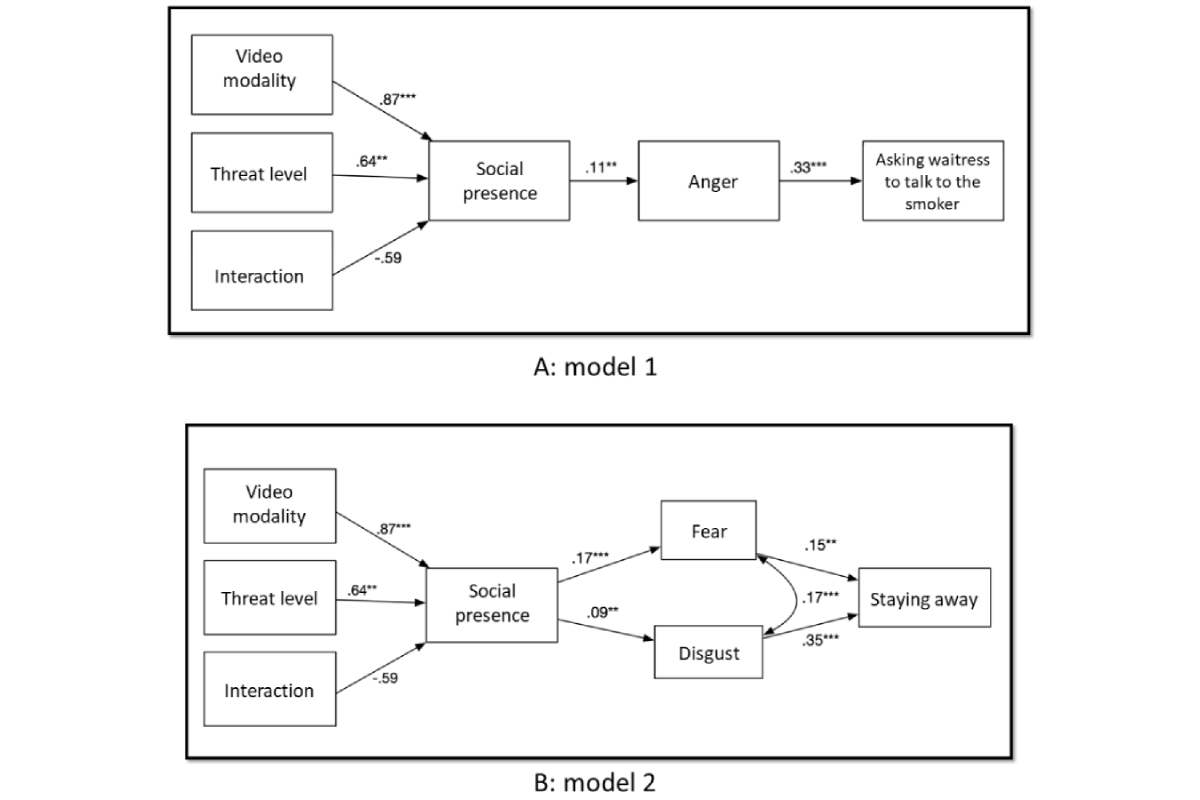
Proposed path models: (A) model 1 with path coefficients and (B) model 2 (adjusted) with path coefficients. **P<.01 and ***P<.001.

Model 1 fit the data well (*χ*^2^_7_=11.1, *P*=.13; CFI=0.91, RMSEA=0.049, and SRMR=0.040). For model 2, the initial hypothesized path model did not fit the data well (*χ*^2^_11_=37.3, *P*<.001; CFI=0.71, RMSEA=0.099, and SRMR=0.077). On the basis of the fit indices of the hypothetical model and modification indices, we revised the model by correlating the error terms of disgust and fear. Finally, all the fit indices of model 2 were satisfied with the conservative criterion (final model: *χ*^2^_10_=14.4, *P*=.16; CFI=0.962, RMSEA=0.042, and SRMR=0.050). Figure 3A and 3B show the path diagrams with a standardized estimation of the final models.

Consistent with hypothesis 1, video modality was a statistically significant predictor of social presence (β*=*.87, SE=.23; *P*<.001). VR induced a higher level of social presence (mean 3.40, SD 1.23) than flat-screen displays (mean 2.85, SD 1.31). The threat level also positively predicted social presence (β*=*.34, SE=.16; *P*=.005). Participants in the high-threat condition perceived higher social presence (mean 3.29, SD 1.28) than those in the low-threat condition (mean 2.95, SD 1.30). Hypothesis 2 was supported.

**Table 1:** Zero-order correlations used in the path analyses^a^ (N=245).

Variable		Video modality		Threat level		Interaction		Social presence		Anger		Fear		Disgust		Stay away		Ask for waitress’s help
**Video modality**																		
	*r*	1	−0.01		0.59		0.22		−0.13		0.07		0.09		0.003		–0.04	
	*P* value	—^b^	.85		<.001		.001		.05		.27		.19		.97		.55	
**Threat level**																		
	*r*	−0.01	1		0.57		0.13		0.06		0.15		0.05		0.06		0.02	
	*P* value	.85	—		<.001		.05		.34		.02		.42		.36		.79	
**Interaction**																		
	*r*	0.59	0.57		1		0.14		0.002		0.19		0.15		0.10		0.02	
	*P* value	<.001	<.001		—		.03		.97		.004		.02		.10		.78	
**Social presence**																		
	*r*	0.22	0.13		0.14		1		0.17		0.25		0.17		0.04		0.10	
	*P* value	.001	.05		.03		—		.009		<.001		.008		.56		.10	
**Anger**																		
	*r*	−0.13	0.06		0.002		0.17		1		0.29		0.63		0.30		0.27	
	*P* value	.51	.34		.97		.009		—		<.001		<.001		<.001		<.001	
**Fear**																		
	*r*	0.07	0.15		0.19		0.25		0.29		1		0.33		0.29		0.05	
	*P* value	.27	.02		.004		<.001		<.001		—		<.001		<.001		.44	
**Disgust**																		
	*r*	0.09	0.05		0.15		0.17		0.63		0.33		1		0.40		0.25	
	*P* value	.19	.42		.02		.008		<.001		<.001		—		<.001		<.001	
**Stay away**																		
	*r*	0.003	0.06		0.10		0.04		0.30		0.29		0.40		1		0.18	
	*P* value	.97	.36		.10		.56		<.001		<.001		<.001		—		.004	
**Ask for waitress’s help**																		
	*r*	−0.04	0.02		0.02		0.10		0.27		0.05		0.25		0.18		1	
	*P* value	.55	.79		.78		.10		<.001		.44		<.001		.004		—	

^a^n=224.

^b^Not applicable.

### Effect of Social Presence on Emotions

In terms of research question 1, the interaction between video modality and threat level was not associated with social presence (β*=−*.59, SE=.32; *P*=.06). Hypothesis 3 stated that social presence positively predicts negative emotions. Consistent with our hypotheses, social presence had a direct influence on anger (β*=*.11, SE=.03; *P*=.008), disgust (β*=*.09, SE=.03; *P*=.007), and fear (β*=*.17, SE=.04; *P*<.001).

### Effect of Emotions on SHS-Related Behaviors

As stated in hypothesis 4, anger positively predicted the intention of asking smokers to stop smoking through the waitress (β*=*.33, SE=.07; *P*<.001). Disgust and fear both positively predicted the intention to stay away (β*=*.35, SE=.06; *P*<.001 for disgust; β*=*.15, SE=.07; *P*=.002 for fear), supporting hypothesis 5. For model 1, the conditional indirect effect of video modality on asking smokers to stop smoking through the waitress was statistically insignificant (95% CI −0.0002 to 0.0632), and the conditional indirect effect of the threat level of asking smokers to stop smoking was statistically insignificant (95% CI −0.002 to 0.049).

### Consideration on Alternative Models

For model 2, the conditional indirect effect of video modality on the intention to stay away was 0.05 (95% CI 0.01-0.09) and the conditional indirect effect of threat level of asking smokers to stop smoking was 0.04 (95% CI –0.004 to 0.067). Because we were unable to confirm the causal relationship between emotion and social presence using cross-sectional data, we also tested the 2 alternative models to further validate the results. To test an alternative model 1, anger was tested as the predictor of social presence. Such an alternative model did not fit the data well (*χ*^2^_7_=40.9, *P*<.001; CFI=0.238, RMSEA=0.141, and SRMR=0.086). The second alternative model treats fear and disgust as the predictors of social presence. The second model also did not fit the data very well (*χ*^2^_9_=98.2, *P*<.001; CFI=0.222, RMSEA=0.201, and SRMR=0.120). To test for an alternative model 2, we then correlated the error term of disgust and fear for model 2, and the fit indices were not satisfactory either (*χ*^2^_8_=73.3, *P*<.001; CFI=0.431, RMSEA=0.183, and SRMR=0.104). As both alternative models did not fit the data well, we can conclude that the proposed models in Figure 2 are more theoretically solid.

## Discussion

### Summary of Findings

Despite the high prevalence of SHS in China, research that focuses on public reactions to SHS in public areas remains scarce. This study explored the potential mediating mechanisms that influence individuals’ responses to SHS risks in public areas based on a college student sample. To our knowledge, this is the first study to explore the health message effect of VR on SHS-related behavioral intentions of nonsmokers. Overall, we found that compared with flat-screen stimuli, the VR stimuli statistically significantly increased participants’ social presence, which in turn influenced negative emotions, and negative emotions statistically significantly mediated the relationships between social presence and SHS-related self-protective behaviors. However, there was no statistically significant interaction effect between threat and video modality on social presence, despite the fact that both threat and video modality had a statistically significant individual effect on social presence. The results of this study suggest that VR is a promising channel for delivering effective health campaigns to motivate nonsmokers’ self-protective behaviors in the SHS environment.

### Principal Findings

This study contributes uniquely to the evidence on the persuasive effects of VR messages on self-protective behaviors related to SHS. We found that the VR-based stimulus induced a higher level of social presence than the flat-screen stimulus. Moreover, threat level, as a contextual factor, also statistically significantly influences social presence. Smoke in this study serves as an important contextual cue that creates an immersive illusion. Therefore, 1 possible explanation for the link between threat level and social presence is that the more smoke is blown by the smokers, the more likely the participants feel they are physically in the SHS environment.

This study also demonstrates the influence of social presence on negative emotions. With a higher level of social presence, participants experienced more negative emotions. This finding supports the relationship between social presence and emotions, as has been confirmed in various contexts. This study also adds to the literature by showing that different negative emotions lead to different behavioral intentions regarding SHS. More specifically, fear and disgust, as avoidance emotions, positively predicted the intention to stay away from the SHS environment, and anger, which is characterized by approach tendency, positively predicted the intention to stop others from smoking through the waitress in public places. When it comes to persuasive health messages, negative emotions could influence viewers’ perception of their future personal risk, and consequently, increase their motivation to adopt self-protective behaviors.

This study has theoretical and practical implications. Theoretically, the results of this study confirm the effect of social presence on negative emotions. We developed a coherent model of SHS-related self-protection behaviors by examining the underlying mechanism linking video modality and threat level to SHS-related self-protection behaviors. We also demonstrated the effectiveness of VR in presenting highly emotionally provocative information. Practically, the findings suggest that VR could be used as an effective tool to facilitate public health campaigns that aim to encourage self-protection behaviors in SHS environments. As the study by Guixeres et al [68] suggested, VR technology provides controllable, multisensory, and interactive 3D computer-generated stimulus environments that offers intervention options that are not possible using traditional methods. By providing an immersive and interactive experience, VR can deepen one’s understanding of the importance and urgency of a particular social issue [44]. VR-based health messages targeting a specific health issue thus merit further research. VR could be strategically used in health communication campaign planning to promote nonsmokers’ self-protective behaviors.

### Comparison With Prior Work

Consistent with the previous literature, social presence is an essential factor that influences individuals’ experiences in a virtual environment. The participants who watched the VR were more likely to feel that they were in the SHS environment than those who watched a video on a computer. This study builds on this immersive effect by adding contextual smoking cues.

Previous literature shows that anger is an emotion that reflects the motivation to respond directly to immediate social threat [69]. On the basis of the results of this study, we concluded that negative emotions play an important role in controlling the adverse effects of SHS. It is recommended that public health message designers consider message features that could effectively induce negative emotions, thereby promoting health behaviors. Our study also calls for the development of culturally tailored health intervention programs in China. In collectivist cultures, it is generally considered impolite or socially inappropriate to stop others smoking directly in public places. As previous research has suggested, collectivists generally prefer maintaining harmony and a nonconfrontational conflict style [70]. Our study highlights the potential of encouraging nonsmokers to ask others for help to communicate with smokers in a collectivist cultural setting. Further research on culturally oriented communication strategies for SHS prevention is needed.

### Limitations

This study had several limitations. First, only participants in VR conditions completed the survey in the laboratory setting, whereas the rest of the participants in the flat-screen conditions completed the web-based experiment. Compared with laboratory experiments, web-based experiments have more environmental variabilities, such as noise, lighting, and technical aspects of the equipment [71] (eg, although the survey cannot be accessed on phones, the variance of the screen size among participants potentially exists, and we cannot ensure the same eye distance from the screen and body posture), which may influence the level of immersion as a predictor of the model.

Second, student samples are generally more homogenous than the general public. Female college students, who constitute a large proportion of the sample, are more likely to have a higher level of knowledge about SHS [72], as it has been found in previous literature that female individuals with at least college education or nonsmokers were more likely to choose restaurants and bars with restrictions on smoking [73]. College students are also less likely to be exposed to SHS, as Chinese college students are required to live on campus, and college students are generally nonsmokers or lighter smokers.

Third, we did not measure whether the participants have gambling disorder problems. Because the experiment stimuli contain the scene of smokers playing poker cards, it is possible that participants who are addictive to gambling are less sensitive to smoking in the VR environment. Finally, there could be unmeasured variables and other confounding factors, such as participants’ previous VR use and attitude toward smoking, which could have influenced the results. Future studies should also consider these factors.

### Future Directions

Further research is needed to determine whether the results of this study can be replicated in other age groups. As demonstrated in this study, VR could be an effective way to enhance the behavioral intention of stopping others smoking in public areas among college students. However, it is unknown whether these results can be replicated using different sampling frames. More augmented reality–based and VR-based health interventions that target specific populations are needed. Prospective studies examining the effect of VR on health behavioral outcomes and providing conclusive evidence of the mediating mechanisms are also recommended. In addition, future research can be built from this study by examining the effects of different message delivery methods.

### Conclusions

In conclusion, our study demonstrates that social presence and negative emotions are 2 important mediators underlying the relationship between video modality and behavioral intention regarding SHS in a 360°-video VR setting. More specifically, we found that (1) smoke as a contextual risk factor increases social presence alongside video modality and (2) negative emotion can be a key factor relating to behavioral intention. Health practitioners are recommended to incorporate VR technologies into more public health strategies and practices.

## Abbreviations

CFI: comparative fit index
HMD: head-mounted display
IVE: immersive virtual environment
RMSEA: root mean square error of approximation
SHS: secondhand smoke
SRMR: standardized root mean square residual
VR: virtual reality

## Data Availability

The data sets generated during and analyzed during this study are available from the corresponding author upon reasonable request.
